# The Prognostic Value of Right Atrial and Right Ventricular Functional Parameters in Systemic Sclerosis

**DOI:** 10.3389/fcvm.2022.845359

**Published:** 2022-03-17

**Authors:** Jacqueline L. Vos, Steele C. Butcher, Federico Fortuni, Xavier Galloo, Laura Rodwell, Madelon C. Vonk, Jeroen J. Bax, Sander I. van Leuven, Jeska K. de Vries-Bouwstra, Miranda Snoeren, Saloua El Messaoudi, Nina A. Marsan, Robin Nijveldt

**Affiliations:** ^1^Department of Cardiology, Radboud University Medical Center, Nijmegen, Netherlands; ^2^Department of Cardiology, Leiden University Medical Center, Leiden, Netherlands; ^3^Department of Cardiology, Royal Perth Hospital, Perth, WA, Australia; ^4^Department of Cardiology, San Giovanni Battista Hospital, Foligno, Italy; ^5^Section Biostatistics, Department for Health Evidence, Radboud Institute for Health Sciences, Nijmegen, Netherlands; ^6^Department of Rheumatology, Radboud University Medical Center, Nijmegen, Netherlands; ^7^Department of Rheumatology, Leiden University Medical Center, Leiden, Netherlands; ^8^Department of Radiology and Nuclear Medicine, Radboud University Medical Center, Nijmegen, Netherlands

**Keywords:** systemic sclerosis, right ventricular function, right atrial strain, feature tracking, prognosis

## Abstract

**Introduction:**

Right ventricular (RV) function is of particular importance in systemic sclerosis (SSc), since common SSc complications, such as interstitial lung disease and pulmonary hypertension may affect RV afterload. Cardiovascular magnetic resonance (CMR) is the gold standard for measuring RV function. CMR-derived RV and right atrial (RA) strain is a promising tool to detect subtle changes in RV function, and might have incremental value, however, prognostic data is lacking. Therefore, the aim of this study was to evaluate the prognostic value of RA and RV strain in SSc.

**Methods:**

In this retrospective study, performed at two Dutch hospitals, consecutive SSc patients who underwent CMR were included. RV longitudinal strain (LS) and RA strain were measured. Unadjusted cox proportional hazard regression analysis and likelihood ratio tests were used to evaluate the association and incremental value of strain parameters with all-cause mortality.

**Results:**

A total of 100 patients (median age 54 [46–64] years, 42% male) were included. Twenty-four patients (24%) died during a follow-up of 3.1 [1.8–5.2] years. RA reservoir [Hazard Ratio (HR) = 0.95, 95% CI 0.91–0.99, *p* = 0.009] and conduit strain (HR = 0.93, 95% CI 0.88–0.98, *p* = 0.008) were univariable predictors of all-cause mortality, while RV LS and RA booster strain were not. RA conduit strain proved to be of incremental value to sex, atrial fibrillation, NYHA class, RA maximum volume indexed, and late gadolinium enhancement (*p* < 0.05 for all).

**Conclusion:**

RA reservoir and conduit strain are predictors of all-cause mortality in SSc patients, whereas RV LS is not. In addition, RA conduit strain showed incremental prognostic value to all evaluated clinical and imaging parameters. Therefore, RA conduit strain may be a useful prognostic marker in SSc patients.

## Introduction

Systemic sclerosis (SSc) is a systemic immune-mediated disease that is characterized by vasculopathy, inflammation and fibrosis of the skin and internal organs. Pulmonary arterial hypertension (PAH) and primary heart disease are among the leading causes of death, highlighting the importance of detecting cardiac involvement in patients with SSc (1). Overt cardiac manifestations of SSc range from inflammatory diseases such as myo- or pericarditis to conduction defects, heart failure, and PAH (2–4). Although cardiac disease is common in SSc, many cardiac manifestations remain subclinical, and reported prevalence highly depends on the sensitivity of the diagnostic method (3, 4). However, prevalence increases with growing disease duration (5). Early detection of cardiac complications could influence treatment decisions in patients, hence there is an unmet need for safe and easily applicable diagnostic measurements in SSc.

Over previous decades, cardiac magnetic resonance imaging (CMR) studies have provided insight into the etiology of the wide range of cardiac alterations observed in SSc. CMR studies, being able to characterize cardiac tissue and coronary ischemia, suggest that cardiac fibrosis, inflammation and vasculopathy play a pivotal role (3). Even in asymptomatic patients with normal echocardiographic parameters, CMR can identify a pattern of local or diffuse fibrosis and low-grade inflammation (6–8). Some asymptomatic SSc patients even show a pattern suggestive of myocarditis, which normalizes after immunosuppressive treatment (9). These findings may represent an early stage of myocardial involvement, compared to the later stages of the diffuse and extended fibrosis which have been described in autopsy studies (10, 11). Therefore, CMR is currently used most often in SSc patients with suspected cardiac involvement. Furthermore, CMR is considered the gold standard for the evaluation of right ventricular (RV) function (12). Considering that the RV can be affected by several common SSc manifestations leading to increased RV afterload [such as interstitial lung disease (ILD), PAH, and primary left-sided cardiac disease], the assessment of RV function is of utmost importance in patients with SSc (1, 12).

Despite the growing evidence of the importance of SSc cardiac manifestations, in clinical practice it remains challenging to distinguish which patients should be monitored more closely. CMR feature tracking is a promising technique measuring strain on standard cine images, detecting subtle changes in cardiac function (13). Thus, right atrial (RA) and RV strain may be more sensitive tools to evaluate RA and RV function than standard volumetric measures or ejection fraction (EF). However, their prognostic value is still unknown. Therefore, the aim of our study was to evaluate the prognostic value of RA and RV strain in SSc patients, and to evaluate their incremental prognostic value to known clinical and imaging parameters.

## Materials and Methods

### Study Population

This study was performed at two Dutch tertiary referral centers for SSc patients [Radboud University Medical Center (Nijmegen)] and [Leiden University Medical Center (Leiden)] and with standardized healthcare programs and annual disease activity and complication screening and multidisciplinary team care. Consecutive patients (>18 years), diagnosed with SSc, fulfilling the 2013 American College of Rheumatology and European League Against Rheumatism criteria (14), and with a clinical indication for CMR, were included (January 2009 to September 2020). For the Radboud University Medical Center cohort, patients were retrospectively included, whereas for the Leiden University Medical Center cohort, patients were prospectively included as part of the ongoing cohort study on systemic sclerosis [Combined Care in SSc cohort; CCIS (15)]. Clinical data, including medical history, clinical presentation, modified Rodnan Skin Score (mRSS), chest computed tomography (CT), and pulmonary function tests [including the forced vital capacity (FVC) % of predicted, and the diffusing capacity of the lungs for carbon monoxide (DLCO) % of predicted] were collected using medical records or extracted from the CCIS. In addition, echocardiographic data was gathered. Diastolic function was assessed by measuring early (E) and late/atrial (A) velocities, using pulsed wave Doppler-imaging in the apical 4-chamber view, and pulsed-wave tissue Doppler for early diastolic velocities at the lateral mitral annulus (e’ lateral). E/A and E/e’ lateral ratios were calculated. The RV systolic pressure (RVSP) was calculated by adding the Bernoulli equation derived pressure gradient from the maximum tricuspid regurgitation velocity to the estimated RA pressure (16). The study was performed according to the declaration of Helsinki and was approved by the Local Institutional Medical Ethics Committees. Written informed consent was waived by the local institutional review board of the Radboud University Medical Center, and written informed consent was obtained for the patients included in the Combined Care in SSc cohort study at the Leiden University Medical Center.

### Follow-Up and the Primary Endpoint

Follow-up data were collected, the start of follow-up was defined as the date of the CMR, and the end of follow-up as the last date of contact (till July 2021). There were no patients lost to follow-up. The primary endpoint was defined as all-cause mortality.

### Cardiovascular Magnetic Resonance Acquisition and Analysis

Patients were scanned on commercially available 1.5T CMR scanners (Gyroscan ACS-NT/Intera MRI system; Philips Medical Systems, Best, Netherlands, or Siemens Avanto; Siemens Healthcare, Erlangen, Germany), or a 3.0-T CMR scanner (Ingenia MRI system; Philips Medical Systems, Best, Netherlands). Standard cine images in long- and short-axis views were acquired during expiratory breath holds using a balanced steady-state free precession sequence. Left ventricular (LV) and RV mass, volumes and EF were measured on the consecutive short-axis cine images (from the base to apex). Late gadolinium enhancement (LGE) imaging was performed 10–15 min after administration of a gadolinium-based contrast agent (Gadovist 0.1 mmol/kg or Dotarem 0.2 mmol/kg), and LGE presence and localization were visually evaluated.

#### Feature-Tracking Derived Right Atrial, Right Ventricular, and Left Ventricular Strain Analysis

**FIGURE 1:**
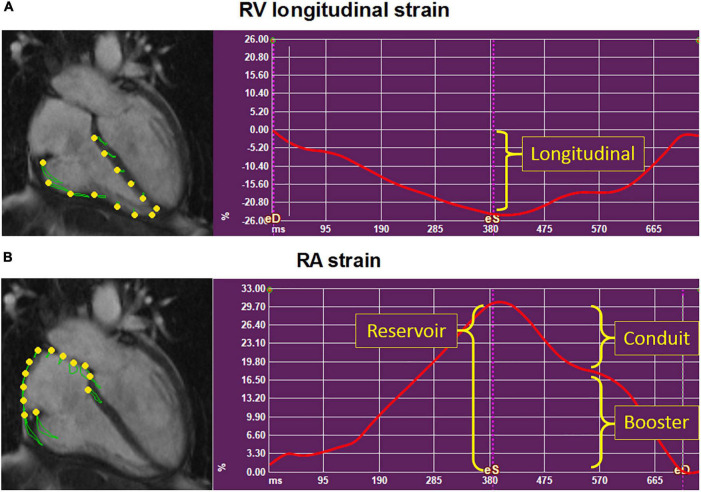
Depiction of the right ventricular and right atrial strain parameters. Example of the strain parameters (*y*-axis, %) in time (*x*-axis, ms) in a systemic sclerosis patient, using Medis Qstrain software. Right ventricular (RV) longitudinal strain **(A)** and right atrial (RA) strain **(B)** were measured on the long-axis 4-chamber cine images. **(B)** RA reservoir strain measures RA filling of blood from the central venous return during RV systole. RA conduit strain measures the passive emptying of blood from the RA into the RV. RA booster strain measures the active filling of blood from the RA to the RV: The RA contraction. RA, right atrial; RV, right ventricular.

Right atrial and RV strain were measured on the standard 4-chamber long-axis cine images using Medis Qstrain software (Medis Medical Imaging Systems, version 2.0). LV global longitudinal strain (LS) was measured on the 2-, 3,- and 4-chamber cine images. RA, RV, and LV endocardial contours were manually drawn in the end-systolic and end-diastolic phases, and the software automatically tracks the contours in the consecutive frames. LV and RV LS, RA reservoir (passive RA filling, collecting the central venous return), conduit (passive filling of blood from the RA to the RV, during the early, passive diastolic phase), and booster strain (RA contraction, during the late, active, diastolic phase) were measured on the 4-chamber long-axis cines. RV and RA strain parameters are illustrated in Figure 1. If patients had atrial fibrillation during the CMR, since there is no atrial contraction, RA booster strain was left out. RA volumes and EF, using the biplane Simpson’s area-length method (17), are automatically generated by the Qstrain software.

### Statistical Analysis

Variables are displayed as number (percentage), mean ± standard deviation or median [interquartile range], as appropriate. To evaluate whether RA and RV functional parameters were correlated with signs of elevated pulmonary artery pressure (tricuspid regurgitation velocity and estimated RVSP), or SSc complications that can impact RV afterload, such as the severity of ILD (FVC % of predicted), and/or pulmonary hypertension (DLCO % of predicted), or left-sided heart disease (LVEF, LV LS, and E/e’ lateral ratio), the Pearson correlation coefficient was evaluated. Kaplan-Meier survival curves were estimated for strain parameters using the medians, and differences between groups were assessed by the log-rank test. To evaluate the prognostic value of clinical and imaging (including RV and RA strain) parameters, unadjusted cox proportional hazards regression analyses were performed to estimate the hazard ratio (HR) and 95% confidence interval (CI). A sub-analysis was performed in SSc patients with a still preserved RV systolic function (RVEF ≥ 50%), to evaluate whether strain parameters can be of predictive value even before RV functional decline. All numeric variables (including the strain parameters) were included as continuous variables in the cox proportional hazard regression analyses. The incremental prognostic value of univariable significant strain parameters were evaluated by testing the likelihood ratio (LR) to predictive covariates. All statistical analyses were performed using SPSS (version 25). A two-sided *p*-value less than 0.05 was considered statistically significant.

## Results

### Patient Characteristics

A total of 100 patients (median age 54 [46–64] years, 42% male) were included. Baseline characteristics are summarized in Table 1. Half of the patients had diffuse systemic sclerosis, and the median non-Raynaud’s phenomenon duration was 3 [1–8] years. Pulmonary and gastro-intestinal involvement were present in approximately half of patients. Twenty-eight percent of patients had a history of cardiovascular disease, most commonly atrial fibrillation (paroxysmal or permanent, *n* = 15) or known coronary artery disease (*n* = 14). At the time of the CMR, two patients (2%) had PAH and were treated with dual therapy (phosphodiesterase inhibitors and endothelin receptor antagonists). During follow-up five patients (5%) developed new-onset PAH. All were treated with mono- (*n* = 2) or dual therapy (*n* = 3) with phosphodiesterase inhibitors and/or endothelin receptor antagonists. Most patients presented with dyspnea (54%, NYHA class > II in 24%) and/or chest pain (24%). The indication for CMR was most often for suspected myocarditis (41%) or new onset heart failure (19%), followed by screening for lung- or autologous stem cell transplantation (10%), suspicion of coronary artery disease (10%), unexplained elevated Hs-troponin T (9%), and tachy- or brady-arrhythmias (7%, Table 2).

**TABLE 1:** Baseline characteristics of systemic sclerosis patients.

	Patients (*n* = 100)
Age (years)	54 [46–64]
Male (n)	42 (42%)
BSA (m^2^)	1.8 ± 0.2
BMI (kg/m^2^)	23 [21–25]
**Cardiac history**	
Atrial fibrillation (paroxysmal or permanent) (n)	15
Known coronary artery disease (n)	14
Previous myocardial infarction (n)	8
Pacemaker, CRT and/or ICD (n)	5
**Cardiovascular risk factors**	
Smoking (n)	
Never	46 (46%)
Current	15 (15%)
Previous	39 (39%)
Hypertension (n)	21 (21%)
Hypercholesterolemia (n)	10 (10%)
Diabetes Mellitus (n)	6 (6%)
**Systemic sclerosis characteristics**	
Diffuse systemic sclerosis (n)	50 (50%)
Raynaud’s phenomenon (n)	97 (97%)
Raynaud’s phenomenon duration (years)	6 [2–15]
Non-Raynaud’s phenomenon duration (years)	3 [1–8]
Modified Rodnan Skin Score (points)	6 [2–17]
Digital ulcers (n)	16 (16%)
Gastro-intestinal involvement (n)	54 (54%)
Pulmonary arterial hypertension (n)	2 (2%)
**Clinical presentation**	
Chest pain (n)	24 (24%)
Dyspnea (n)	54 (54%)
Syncope (n)	8 (8%)
NYHA class > II (n)	24 (24%)
**Medical therapy**	
Diuretics (n)	6 (6%)
Calcium channel blocker (n)	43 (43%)
Corticosteroids (n)	35 (35%)
Cyclophosphamide (n)	6 (6%)
Methotrexate (n)	12 (12%)
Azathioprine (n)	5 (5%)
Mycophenolate mofetil (n)	18 (18%)
**Laboratory findings**	
Creatinine	71 [57–89]
Estimated GFR (ml/min/1.73 m^2^)	88 [60–90]
C-reactive protein (mg/l)	5 [1–12]
Elevated Hs-troponin T (>14 ng/L) (n = 66)	22 (22%)
**Pulmonary functional testing (n = 95)**	
Forced vital capacity of predicted (%)	88 [62–101]
DLCO of predicted (%)	56 [43–68]
Reduced DLCO of predicted (≤70%)	74 (78%)
**Computed tomography scan (n = 90)**	
Presence of interstitial lung disease (n)	52 (58%)
Ground glass (n)	33 (37%)
Honeycombing (n)	6 (7%)
Fibrosis (n)	40 (44%)
**Transthoracic echocardiography (n = 99)**	
E/A ratio	1.1 [0.8–1.3]
E/e’ lateral ratio	10 [7–14]
TR velocity (m/s) (n = 86)	2.5 ± 0.5
Estimated RVSP (mmHg) (n = 86)	28 [22–35]

*Data is presented as mean ± standard deviation, median [interquartile range] or number (%). CRP, C-reactive protein; CRT, cardiac resynchronization therapy; DLCO, diffusing capacity of the lungs for carbon monoxide; GFR, glomerular filtration rate; ICD, implantable cardioverter defibrillator; NYHA, New York Heart Association; TR, tricuspid regurgitation; RVSP, right ventricular systolic pressure.*

**TABLE 2:** CMR characteristics of systemic sclerosis patients.

	Patients (*n* = 100)
**Indication for CMR (n)**	
Myocarditis	41 (41%)
New onset heart failure	19 (19%)
Suspicion of CAD	10 (10%)
Unexplained elevated Hs-troponin T	9 (9%)
Screening for lung or autologous stem cell transplantation	10 (10%)
Tachy- or brady-arrhythmia’s	7 (7%)
**LV- and RV volumes**	
LVEDV (mL)	156 [134–185]
LVEDV indexed (mL/m^2^)	90 [74–104]
LVESV (mL)	64 [48–86]
LVESV indexed (mL/m^2^)	35 [26–49]
LVEF (%)	60 [51–66]
LV mass (g)	106 ± 29
LV mass indexed (mL/m^2^)	59 ± 15
RVEDV (mL)	154 [128–185]
RVEDV indexed (mL/m^2^)	86 [71–105]
RVESV (mL)	74 [55–94]
RVESV indexed (mL/m^2^)	41 [31–54]
RVEF (%)	52 [47–59]
RV mass (g)	21 ± 7
RV mass indexed (g/m^2^)	11 ± 3
RA maximal volume indexed (mL/m^2^)	48 ± 19
RA EF	46 ± 13
**LGE presence (n = 95)**	20 (21%)
Ischemic (focal subendocardial or transmural)	7 (8%)
Non-ischemic (epi, mid- or patchy)	10 (50%)
Insertion RV	3 (3%)
Pericardial	1 (1%)
**Strain measurements**	
LV global longitudinal strain (%)	−21 ± 6
RV global longitudinal strain (%)	−26 ± 7
RA reservoir strain (%)	36 ± 12
RA conduit strain (%)	18 ± 9
RA booster strain (%)	18 ± 8

*Data is presented as mean ± standard deviation, median [interquartile range] or number (%). CAD, coronary artery disease; CMR, cardiac magnetic resonance; EDV, end-diastolic volume; ESV, end-systolic volume; EF, ejection fraction; LGE, late gadolinium enhancement; LV, left ventricular; RA, right atrial; RV, right ventricular.*

### Cardiovascular Magnetic Resonance Parameters

Cardiovascular magnetic resonance characteristics are shown in Table 2. Both median LVEF {60 [interquartile range (IQR) 51–66]%} and RVEF (51 [IQR 47–59]%) were preserved. However, 21% of patients had reduced LVEF (<50%), and 39% of patients had a reduced RVEF (<50%). LGE was present in 20 patients (21%). The most common LGE pattern was non-ischemic (*n* = 10, 50%), followed by ischemic (*n* = 7, 35%). Three patients had LGE at the insertion of the RV and one patient had pericardial enhancement. Two patients (2%) had atrial fibrillation during the CMR scan, the cine images were of sufficient quality for both. Feature tracking RV (*n* = 99) and RA strain (*n* = 98) analysis were feasible in almost all patients.

### Correlation of Imaging Parameters With Increased Right Ventricular Afterload Parameters

To evaluate whether RA and RV functional parameters were correlated with indices of elevated pulmonary artery pressure (tricuspid regurgitation velocity and estimated RVSP) or with conditions which can affect RV afterload, such as the severity of ILD (FVC % of predicted) and/or pulmonary hypertension (DLCO % of predicted), and left-sided heart disease (LVEF, LV LS, and E/e’ lateral), the Pearson correlation coefficient was assessed (Table 3). All RA and RV imaging parameters were correlated with LVEF and LV LS. Both RA reservoir, RA conduit strain and RV LS were correlated with the E/e’ lateral ratio. RVEF, RV LS, and RA conduit strain showed significant correlations with DLCO. Interestingly, none of the imaging parameters were associated with indices of elevated pulmonary artery pressures, except for RA conduit strain (see Table 3 for values).

**TABLE 3:** Pearson correlation coefficients of right atrial and right ventricular functional parameters with estimated pulmonary arterial pressure on echocardiography, DLCO and left ventricular function.

	TR velocity (m/s)	Estimated RVSP (mmHg)	FVC of predicted (%)	DLCO of predicted (%)	LVEF (%)	LV longitudinal strain (%)	E/e’ ratio
RVEDV indexed (mL/m^2^)	0.129	0.122	0.019	0.111	−0.229*	0.207*	−0.016
RVEF (%)	−0.158	−0.183	0.220*	0.332*	0.617*	−0.578*	−0.187
RA maximal volume indexed (mL/m^2^)	0.052	0.017	0.162	0.101	−0.243*	0.337*	0.121
RV global longitudinal strain (%)	0.179	0.205	−0.235*	−0.284*	−0.456*	0.460*	0.215*
RA reservoir strain (%)	−0.119	−0.118	0.064	0.191	0.393*	−0.482*	−0.316*
RA conduit strain (%)	−0.299*	−0.276*	0.172	0.313*	0.347*	−0.430*	−0.368*
RA booster strain (%)	0.117	0.095	−0.093	−0.019	0.211*	−0.232*	−0.035

DLCO, diffusing capacity of the lungs for carbon monoxide; EDV, end diastolic volume; EF, ejection fraction; FVC, forced vital capacity; LV, left ventricular; RA, right atrial; RV, right ventricular; RVSP, right ventricular systolic pressure; TR, tricuspid regurgitation. *p < 0.05. Significant p-values (p < 0.05). Significant p-values (p < 0.05) are indicated in bold.

### Association of Imaging Parameters With All-Cause Mortality

**FIGURE 2:**
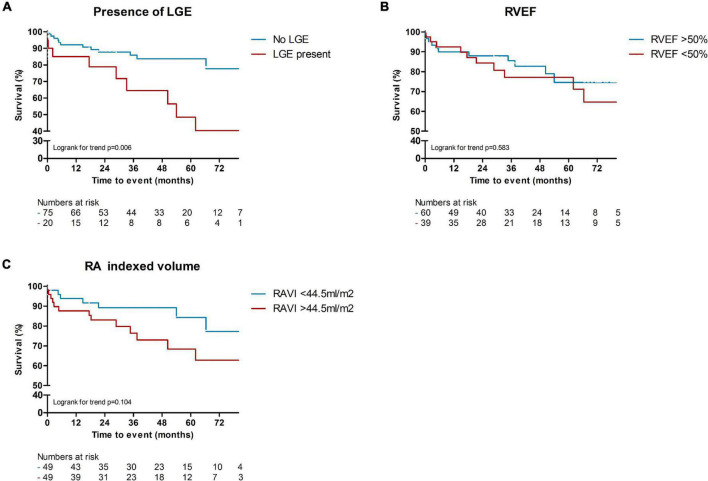
Kaplan-Meier survival analysis of LGE, RVEF, and RA indexed volume. Presence of LGE was associated with higher rates of all-cause mortality, compared to patients without any LGE [*p* = 0.006, **(A)**]. Compared to SSc patients with preserved RVEF (>50%), patients with reduced RVEF (<50%), did not have a worse prognosis [*p* = 0.583, **(B)**]. A higher RA indexed volume (>44.5 ml/m^2^) was also not significantly associated with worse all-cause mortality [*p* = 0.104, **(C)**]. LGE, late gadolinium enhancement; RVEF, right ventricular ejection fraction; RA (VI), right atrial (volume indexed).

**FIGURE 3:**
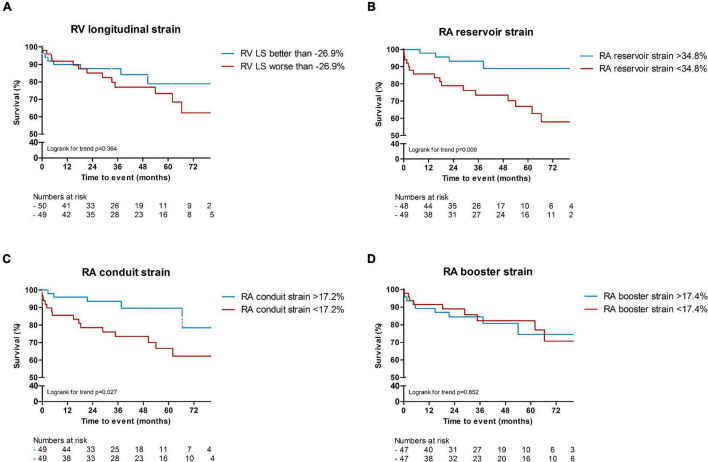
Kaplan Meier survival analysis of RV LS, RA reservoir and conduit strain. RV longitudinal strain above or below the median was not associated with a difference in all-cause mortality [*p* = 0.364, **(A)**]. RA reservoir (<34.8%), and RA conduit strain below the median (<17.2%) were associated with higher all-cause mortality rates compared to patients with RA reservoir or conduit strain above the median [*p* = 0.009, and *p* = 0.027, **(B,C)**]. Differences in median RA booster strain was not associated with differences in all-cause mortality [*p* = 0.852, **(D)**]. RV, right ventricular; LS, longitudinal strain; RA, right atrial.

In total, 24 patients (24%) died during a follow-up of 3.1 [1.8–5.2] years. The cause of death was cardiac related in 38% of patients [heart failure (*n* = 5) and sudden cardiac death (*n* = 4)], severe ILD in 17% (*n* = 4) and PAH in 8% (*n* = 2). In 25% it was related to multi-organ failure [septic shock post stem cell transplantation (*n* = 1), ileus in a patient with severe ILD and heart failure (*n* = 1), myocarditis, pneumonia and renal insufficiency (*n* = 1), myositis and spontaneous severe hemorrhage in a cachectic patient (*n* = 1), cachectic patient with gastro-intestinal disease and ILD (*n* = 1), severe arterial disease in a patient with suspected PAH and renal failure (*n* = 1)]. One patient died of a renal crisis, one of a malignancy, and it is unknown in one patient. Patients with LGE had a significantly worse outcome compared to patients without any LGE (*p* = 0.006, Figure 2A). Compared to SSc patients with preserved RVEF, patients with reduced RVEF (<50%), did not have a worse prognosis, in respect to all-cause mortality (*p* = 0.583, Figure 2B). In addition, RA indexed volume above the median (>44.5 ml/m^2^) was not associated with a higher death rate (*p* = 0.104, Figure 2C). Regarding strain parameters, RV LS was not associated with differences in all-cause mortality (*p* = 0.364, Figure 3A). Interestingly, both RA reservoir (<34.8%), and RA conduit strain below the median (<17.2%) were associated with higher all-cause mortality rates compared to patients with RA reservoir or conduit strain above the median (*p* = 0.009, and *p* = 0.027, Figures 3B,C, respectively). Finally, RA booster strain was not associated with differences in all-cause mortality (*p* = 0.852, Figure 3D).

### Prognostic Value of Right Ventricular and Right Atrial Strain to Predict All-Cause Mortality

Right atrial reservoir [Hazard Ratio (HR) = 0.95, 95% CI 0.91–0.99, *p* = 0.009] and conduit strain (HR = 0.93, 95% CI 0.88–0.98, *p* = 0.008) were univariable predictors of all-cause mortality (included as continuous variables, Table 4). RV LS and RA booster strain were not associated with mortality. RA maximum volume indexed was associated with all-cause mortality. Surprisingly, none of the LV- or RV volumes or EF were associated with mortality. To assess the predictive value of different strain parameters in preserved RV systolic function (RVEF ≥ 50%, *n* = 60), a sub-analysis was performed. Both RA reservoir (HR = 0.95, 95% CI 0.91–0.99, *p* = 0.010) and conduit strain (HR = 0.92, 95% CI 0.87–0.98, *p* = 0.008) remained to be of prognostic significance, whereas RV LS (HR = 1.03, 95% CI 0.98–1.09, *p* = 0.248) and RA booster strain were not (HR = 0.99, 95% CI 0.93–1.06, *p* = 0.816). Presence of any LGE, and presence of non-ischemic LGE were related to mortality. Of the clinical covariates, male sex, atrial fibrillation, and a NYHA class of > II were univariable predictors of all-cause mortality (Table 4). Since previous research showed that RA and RV strain values differ between males and females (18), a sub-analysis was performed to evaluate whether the correlation of RA strain with all-cause mortality differed between the sexes. In female patients (*n* = 58), RA reservoir and conduit strain were predictors of all-cause mortality (*n* = 9, data not shown). Interestingly, in male patients (*n* = 42) both RA reservoir and conduit strain were not associated with all-cause mortality (RA reservoir strain: HR = 0.98, 95% CI 0.94–1.03, *p* = 0.421; and RA conduit strain: HR = 0.97, 95% CI 0.90–1.04, *p* = 0.376).

**TABLE 4:** Univariable association with all-cause mortality.

	Systemic sclerosis patients (*n* = 100)	
	
	Hazard ratio (95% CI)	p-value
Age (years)	1.02 (0.99−1.05)	0.189
Sex (male)	2.65 (1.15−6.08)	**0.021**
Diffuse systemic sclerosis	0.91 (0.41−2.03)	0.810
Age of diagnosis >60 years	1.44 (0.57−3.67)	0.454
Atrial fibrillation	4.02 (1.72−9.42)	**0.001**
FVC of predicted (%)	0.98 (0.97−1.00)	0.058
DLCO of predicted (%)	0.97 (0.94−1.00)	0.069
Pulmonary involvement	1.47 (0.62−3.48)	0.379
Known coronary artery disease	2.63 (0.96−7.23)	0.061
NYHA class > II	3.38 (1.45−7.86)	**0.005**
Estimated RV systolic pressure (mmHg)*	1.03 (1.00−1.06)	0.068
**Baseline CMR parameters**		
LV end-diastolic volume-indexed (mL/m2)	1.00 (0.98−1.02)	0.966
LVEF (%)	0.97 (0.94−1.00)	0.075
RV end-diastolic volume-indexed (mL/m^2^)	1.00 (0.99−1.02)	0.742
RVEF (%)	0.99 (0.95−1.03)	0.531
RA maximum volume-indexed (mL)	1.02 (1.00−1.04)	**0.031**
RA EF (%)	0.97 (0.94−1.01)	0.130
Presence of LGE	3.42 (1.42−8.28)	**0.006**
Presence of non-ischemic LGE	3.59 (1.43−9.01)	**0.007**
**Strain measurements**		
RV global longitudinal strain (%)	1.03 (0.98−1.09)	0.248
RA reservoir strain (%)	0.95 (0.91−0.99)	**0.009**
RA conduit strain (%)	0.93 (0.88−0.98)	**0.008**
RA booster strain (%)	1.01 (0.95−1.07)	0.799

CMR, cardiac magnetic resonance; DLCO, diffusing capacity of the lungs for carbon monoxide; EF, ejection fraction; FVC, forced vital capacity; LGE, late gadolinium enhancement; LV, left ventricular; RA, right atrial; RV, right ventricular. *Available in 86 patients. Significant p-values (p < 0.05) are indicated in bold.

To evaluate the incremental prognostic value of RA reservoir and conduit strain, an LR test was performed with the clinical and imaging covariates which were univariable predictors of all-cause mortality. RA reservoir strain proved to be of incremental value to male sex (LR χ^2^ = 5.34, *p* = 0.021), atrial fibrillation (LR χ^2^ = 3.86, *p* = 0.049), NYHA class > II (LR χ^2^ = 3.99, *p* = 0.046), but not on RA indexed volume (LR χ^2^ = 3.40, *p* = 0.065) or the presence of LGE (LR χ^2^ = 3.05, *p* = 0.081). RA conduit strain was of incremental predictive value to all clinical and imaging parameters (male sex LR χ^2^ = 5.81, *p* = 0.016; atrial fibrillation LR χ^2^ = 5.68, *p* = 0.017; NYHA class > II LR χ^2^ = 4.53, *p* = 0.033; LGE presence LR χ^2^ = 6.34, *p* = 0.012; and RA indexed volume LR χ^2^ = 5.97, *p* = 0.015).

## Discussion

This study evaluates the prognostic value of CMR derived RA and RV strain in SSc patients. RA reservoir and conduit strain were predictors of all-cause mortality, even in preserved RV systolic function (RVEF ≥ 50%), whereas RA booster and RV LS were not. In addition, RA conduit strain was of incremental value to clinical and imaging parameters, such as sex, atrial fibrillation, NYHA class, indexed RA volume and presence of LGE. Therefore, CMR derived RA conduit strain may be useful for identifying SSc patients at higher risk who may need a closer follow-up.

Systemic sclerosis is accompanied by a high morbidity and mortality. In the previous two decades it has become clear that cardiac involvement, which has an asymptomatic course in a substantial part of patients, is one of the main contributors (1). Therefore, it is of major significance to identify and diagnose patients early in the course of the disease. LGE and parametric mapping were found to be of prognostic value in SSc patients (19), however, further data about the prognostic value of CMR is lacking. Evidence showing the importance of non-invasive RV imaging for prognostication in conditions where the RV afterload may be increased is growing (20). Feature tracking might be a promising marker for early detection of subtle changes in cardiac function in several conditions (13). A recent study shows that, although EF was similar, RV LS was impaired in asymptomatic SSc patients compared to healthy controls (21). Although promising, the prognostic value of feature tracking derived RV or RA strain in SSc patients has yet to be evaluated. This study emphasizes the need for detailed functional evaluation of the RV and RA, using feature tracking software. This software is easy to apply on the standard cine images. Therefore, it has the potential to be readily incorporated in daily clinical practice to refine clinical management.

In this study, we focused on the right side of the heart, as SSc can secondarily affect the RV. Various common SSc manifestations, such as ILD, left sided heart disease and/or PAH (22), can lead to pulmonary hypertension and thus increased RV afterload. Extra attention is paid to RV function in SSc patients, since in pulmonary hypertension RV adaptation is key for survival (23, 24), and patients with SSc associated PAH are known to have the worst prognosis of all PAH causes (25). There is growing evidence that this poor prognosis is linked to intrinsic RV dysfunction due to primary cardiac involvement (26–29). CMR is currently used more often in SSc patients with suspected cardiac involvement, and is considered the gold standard for analyzing RV function, traditionally by measuring RV volumes and calculating the EF (1, 12). However, at first, RV remodeling, such as increased contractility and hypertrophy, will take place to ensure adequate stroke volume, and a decline in RVEF will not appear until the later stages of heart failure, so other measurements are key to detect cardiac involvement in early stages (23). Feature tracking strain has the potential to detect changes in RV function earlier in the disease. For example, in PAH patients feature tracking RV strain was associated with worse survival, and was found to be impaired even when RVEF was still preserved (30–32). Data about feature tracking derived RA strain are lacking, and in general, RA function is less frequently considered and not part of the standard CMR evaluation. Previous research evaluating RA mechanics shows that even in patients without overt PAH, RA function is impaired in a high proportion of SSc patients (33, 34), and is associated with pulmonary fibrosis and/or elevated pulmonary artery pressures during exercise (35). The RA adaptive mechanisms to elevated RV afterload are elegantly shown in animal studies (36, 37). Although RV systolic function was still preserved in these studies, RV systolic and diastolic pressures were elevated. In response, RA adaptation resulted in increased contractility (higher booster function), and distensibility (higher reservoir function). In contrast, passive filling (RA conduit function) decreased, due to the lower RA-RV pressure gradient. This RA adaptive mechanism, which is confirmed in echocardiographic studies (38, 39), explains the results found in our study. Initial RA adaptation increases RA contractility (booster strain) and distensibility (reservoir strain), until RA compensation falls short, and therefore both decline when the disease progresses. This non-linear relationship with increased RV pressures explains why reservoir and booster strain were not correlated with estimated RV pressures on echocardiography, and why reservoir strain had limited predictive value regarding all-cause mortality. In contrast, RA conduit strain is the first to decline when RV pressures increase, even before RV systolic function declines. This is in agreement with our results, RA conduit strain was correlated to estimated RV systolic pressure, was of predictive value on all-cause mortality even in SSc patients with a still preserved systolic RV function (RVEF > 50%), and of incremental predictive value on all tested clinical and imaging parameters. Therefore, we believe that RA conduit strain may be an early marker for RV dysfunction, and thus a useful parameter for risk stratification and prognostication in SSc patients.

### Limitations

Cardiovascular magnetic resonance parametric mapping has only been performed in recent years, and consequently the association of this parameter with long-term outcome could not be evaluated. In addition, feature-tracking derived RA strain is a novel parameter, and therefore there are no reference data for healthy controls available yet. In a sub-analysis, RA reservoir and conduit strain were predictive of all-cause mortality in females, but not in males. This could be an important finding, however, caution must be applied since the subgroups are small in our study. Since this is a study in SSc patients with a clinical indication for CMR, findings need to be validated in a larger, prospective cohort, also to see whether these results extent to asymptomatic SSc patients as well. All of the echocardiograms have been acquired as part of routine clinical care. Therefore, additional tissue-doppler imaging parameters or speckle tracking strain analysis were frequently missing and could not be evaluated.

## Conclusion

Right atrial reservoir and conduit strain are univariable predictors of all-cause mortality in SSc patients, whereas RV LS is not. In addition, RA conduit strain correlated with estimated pulmonary artery pressures, and was of incremental prognostic value to clinical and imaging parameters. Therefore, RA conduit strain may be a useful prognostic marker in patients with SSc.

## Data Availability Statement

The raw data supporting the conclusions of this article will be made available by the authors, without undue reservation.

## Ethics Statement

The studies involving human participants were reviewed and approved by Research Ethics Committee of the Radboud University Medical Centre and Medical Ethics Committee of Leiden, Hague and Delft. Written informed consent for participation was not required for this study in accordance with the National Legislation and the Institutional Requirements.

## Author Contributions

JV, SB, FF, NM, and RN were involved in the conceptualization of the study. JV, SB, and FF were involved in the acquisition and analysis of the data. JV and LR contributed in the statistical analysis. RN was guarantor of overall content and manuscript writing. All authors were involved in data interpretation, gave intellectual input to improve the manuscript, and read and approved the final version.

## Conflict of Interest

JV-B received consulting fees from AbbVie, Janssen, and Boehringer Ingelheim, and research grants from Roche, Galapagos, and Janssen. MV received research grants from Boehringer Ingelheim, Ferrer, Galapagos, and Janssen, and consulting fees of Boehringer Ingelheim, Corbus, and Janssen. NM received speaker fees from GE Healthcare and Abbott Vascular, and also participated in the Medical Advisory Board of Philips Ultrasound. RN received research grants from Biotronik and Philips, and consulting fees of Sanofi Genzyme and Bayer. The remaining authors declare that the research was conducted in the absence of any commercial or financial relationships that could be construed as a potential conflict of interest.

## Publisher’s Note

All claims expressed in this article are solely those of the authors and do not necessarily represent those of their affiliated organizations, or those of the publisher, the editors and the reviewers. Any product that may be evaluated in this article, or claim that may be made by its manufacturer, is not guaranteed or endorsed by the publisher.
